# Genetic mechanisms of co-emergence of INH-resistant *Mycobacterium tuberculosis* strains during the standard course of antituberculosis therapy

**DOI:** 10.1128/spectrum.02133-23

**Published:** 2024-03-11

**Authors:** Ketema Tafess, Timothy Ting-Leung Ng, Kingsley King-Gee Tam, Kenneth Siu-Sing Leung, Jake Siu-Lun Leung, Lam-Kwong Lee, Hiu Yin Lao, Chloe Toi-Mei Chan, Wing-Cheong Yam, Samson Sai Yin Wong, Terrence Chi-Kwong Lau, Gilman Kit-Hang Siu

**Affiliations:** 1 Department of Applied Biology, School of Applied Natural Sciences, Adama Science and Technology University, Adama, Ethiopia; 2 Institute of Pharmaceutical Sciences, Adama Science and Technology University, Adama, Ethiopia; 3 Department of Health Technology and Informatics, The Hong Kong Polytechnic University, Kowloon, Hong Kong SAR, China; 4 Department of Microbiology, Li Ka Shing Faculty of Medicine, The University of Hong Kong, Pokfulam, Hong Kong SAR, China; 5 Department of Biomedical Sciences, Jockey Club College of Veterinary Medicine and Life Sciences, City University of Hong Kong, Hong Kong, China; Foundation for Innovative New Diagnostics, Geneva, Switzerland

**Keywords:** isoniazid resistance, *Mycobacterium tuberculosis*, whole-genome sequencing, transformation, antituberculosis treatment, protein modeling

## Abstract

The incidence of isoniazid (INH) resistant *Mycobacterium tuberculosis* is increasing globally. This study aimed to identify the molecular mechanisms behind the development of INH resistance in *M. tuberculosis* strains collected from the same patients during the standard course of treatment. Three *M. tuberculosis* strains were collected from a patient before and during antituberculosis (anti-TB) therapy. The strains were characterized using phenotypic drug susceptibility tests, Mycobacterial Interspersed Repeated Unit-Variable-Number Tandem Repeats (MIRU-VNTR), and whole-genome sequencing (WGS) to identify mutations associated with INH resistance. To validate the role of the novel mutations in INH resistance, the mutated *katG* genes were electroporated into a KatG-deleted *M. tuberculosis* strain (GA03). Three-dimensional structures of mutated KatG were modeled to predict their impact on INH binding. The pre-treatment strain was susceptible to INH. However, two INH-resistant strains were isolated from the patient after anti-TB therapy. MIRU-VNTR and WGS revealed that the three strains were clonally identical. A missense mutation (P232L) and a nonsense mutation (Q461Stop) were identified in the *katG* of the two post-treatment strains, respectively. Transformation experiments showed that *katG* of the pre-treatment strain restored INH susceptibility in GA03, whereas the mutated *katG* genes from the post-treatment strains rendered negative catalase activity and INH resistance. The protein model indicated that P232L reduced INH-KatG binding affinity while Q461Stop truncated gene transcription. Our results showed that the two *katG* mutations, P232L and Q461Stop, accounted for the co-emergence of INH-resistant clones during anti-TB therapy. The inclusion of these mutations in the design of molecular assays could increase the diagnostic performance.

**IMPORTANCE:**

The evolution of drug-resistant strains of *Mycobacterium tuberculosis* within the lung lesions of a patient has a detrimental impact on treatment outcomes. This is particularly concerning for isoniazid (INH), which is the most potent first-line antimycobacterial drug. However, the precise genetic factors responsible for drug resistance in patients have not been fully elucidated, with approximately 15% of INH-resistant strains harboring unknown genetic factors. This raises concerns about the emergence of drug-resistant clones within patients, further contributing to the global epidemic of resistance. In this study, we revealed the presence of two novel *katG* mutations, which emerged independently due to the stress exerted by antituberculosis (anti-TB) treatment on a parental strain. Importantly, we experimentally demonstrated the functional significance of both mutations in conferring resistance to INH. Overall, this research sheds light on the genetic mechanisms underlying the evolution of INH resistance within patients and provides valuable insights for improving diagnostic performance by targeting specific mutations.

## INTRODUCTION

The standard treatment for tuberculosis (TB) involves a combination of multiple antituberculosis (anti-TB) drugs. The current 6-month regimen for drug-susceptible TB includes 2 months of intensive phase treatment with isoniazid (INH), rifampicin (RIF), pyrazinamide (PZA), and ethambutol (EMB) followed by 4 months of continuation phases with INH and RIF (1). However, prolonged treatment has led to poor adherence to the regimen, which increases the likelihood of the emergence of drug-resistant *Mycobacterium tuberculosis* strains (2).

Recent studies have reported the emergence of drug-resistant conferring mutations within a patient under standard treatment (3–5). Trauner et al. (3) suggested that the emergence and fixation of drug-resistant conferring mutations depends on the pressure exerted by the drugs. They noted that an accurate dosage of anti-TB drugs substantially clears the bacteria and limits the emergence of drug-resistant strains. In contrast, when the drug pressure declines owing to host-related or programmatic failures, drug-resistant clones would gradually emerge. Strikingly, Liu et al. reported that the microevolution of drug-resistant clones within lung lesions of a patient adversely affects treatment responses reinforcing the above observation (5). The emergence of genetic determinants of drug resistance within patients is becoming a serious concern, particularly for INH due to its complex mechanisms of action and resistance (6).

INH is one of the most powerful first-line antimycobacterial drugs (7). It is a prodrug that requires activation by the bacterial catalase-peroxidase enzyme KatG. The activated INH radicals combine with nicotinamide adenine dinucleotide species (NADH/NAD+) to form INH-NAD adducts that inhibit the biosynthesis of mycobacterial mycolic acid. The drug has pleiotropic effects on both the growing and dormant form of mycobacteria (8). Despite the essential role of INH in anti-TB therapy, the emergence and spreading of INH-resistant *M. tuberculosis* strains become the major challenge for the treatment of TB. The increasing incidence of INH resistance might be attributed to the higher spontaneous mutation rate of *M. tuberculosis* toward INH compared to other drugs (9).

While most of the known INH resistance mechanisms occur through the alteration of genes encoding the activating enzyme or through mutations of other genes that encode the targets of the activated INH, the mechanism of resistance to INH is complex and involves multiple genetic loci (10). Arrays of genetic polymorphisms in genes via *mabA-inhA* promoter, *oxyR-ahpC* intergenic region, *mshA, nat, ndh, kasA, ahp, inhA,* and *katG* have been implicated in INH resistance (11–14). A single point mutation of S315T in *katG* accounts for approximately 60% of INH resistance, while mutations in the regulator and a structural segment of *inhA* contribute to 19% of INH resistance (6). Yet the resistance mechanisms remain elusive with an estimated 15% of INH-resistant strains harboring unknown genetic determinants (15, 16).

Furthermore, with the advancement of deep sequencing technology, it is now possible to identify minor subpopulations of drug-resistant strains in patient specimens. This has raised significant concerns about the emergence of drug-resistant clones in patients who are undergoing standard treatment (17).

This study investigated three *M. tuberculosis* isolates collected from a patient before and during the standard course of TB treatment. The pre-treatment isolate was found to be susceptible to INH, while the two isolates obtained during anti-TB treatment were INH-resistant without any known mutation. Mycobacterial Interspersed Repeated Unit-Variable-Number Tandem Repeats (MIRU-VNTR) typing revealed that all three isolates were clonal. Comparative whole-genome sequencing (WGS) identified two novel mutations in the *katG* gene of the two post-treatment isolates. The functional study of these mutations confirmed their causative role. These findings underscore the need for further studies to elucidate the mechanisms of the emergence of INH resistance within a patient and determine the role of novel mutations through functional genetics.

## MATERIALS AND METHODS

### *M. tuberculosis* clinical isolates

The *M. tuberculosis* isolates were obtained from a 60-year-old male patient diagnosed with pulmonary TB. The initial isolate, labeled as M_11806, was obtained on 28 October 2015, before treatment. Two subsequent isolates, named D1_12327 and D2_12328, were obtained on 11 January and 14 January 2016, respectively, while the patient was undergoing standard anti-TB therapy. Full panel first-line phenotypic drug susceptibility tests (pDST) for the three strains were conducted retrospectively at The University of Hong Kong, while total nucleic acid extracts were then sent to The Hong Kong Polytechnic University for genomic characterization.

In addition, a catalase-negative *M. tuberculosis* isolate (GA03) which exhibited mono-INH resistance (minimum inhibitory concentration = 256 mg/L) and lacked catalase activity, was used as the recipient for cloned *katG* genes in a transformation experiment. Sequencing analysis revealed a truncated *katG* gene with a nonsense mutation (Q434Stop) in the GA03 isolate.

### Phenotypic drug susceptibility tests

Agar proportion susceptibility tests for RIF and INH were performed prospectively in the Public Health Laboratory Centre, the reference laboratory in Hong Kong, according to CSLI guidelines (18). The Bactec MGIT 960 SIRE kit and PZA kit (Becton, Dickinson, Baltimore, MD, USA) were then retrospectively done to determine the first-line drug susceptibilities of the three *M. tuberculosis* isolates according to the previously described protocol (19, 20). The critical concentration values used were 0.1 mg/L and 0.4 mg/mL for INH resistance, 1.0 mg/L for RIF resistance, 5.0 mg/L for EMB resistance, 1.0 mg/L and 4.0 mg/mL for streptomycin (STR) resistance, and 100 mg/L for PZA resistance.

### MIRU-VNTR typing

DNA isolation was conducted using the Roche Cobas Amplicor extraction kit (Roche Diagnostics, USA) as previously described (21). The PCR amplification of the 24 MIRU-VNTR loci was conducted using the primers and PCR conditions provided in MIRU-VNTR (22).

### Whole-genome sequence and bioinformatic analysis

The *M. tuberculosis* strains were initially isolated from respiratory specimens and cultured on either Stonebrink agar (D1_12327) or Lowenstein Jensen slants (M_11896 and D2_12328). Sub-cultures were then performed on 7H11 agar to obtain single colonies. Each strain was individually picked and inoculated into 15 mL of 7H9 broth supplemented with OADC. The cultures were incubated at 37°C with agitation at 120 rpm for 14 days prior to DNA extraction. The phenol-chloroform method was used for isolating genomic DNA as described previously (23–25). The purity and integrity of the genomic DNA were examined using the Qubit HS assay (Thermo Scientific, USA) and 0.8% agarose gel electrophoresis, respectively. The isolated DNA was subjected to QIAseq FX DNA Library Preparation Kit (Qiaen, Hilden, Germany) according to the manufacturer’s instructions, and was sequenced on the Illumina MiSeq platform using 2 × 150 bp version 2 sequencing chemistry (Illumina, San Diego, USA).

Core-genome single nucleotide polymorphism (SNP) phylogeny was constructed to confirm the clonality and the global phylogenetic placement of the three *M. tuberculosis* strains. The detailed methodology was described in Supplemental Materials and Methods.

In addition, a comparative genome analysis was conducted, in which the *M. tuberculosis* H37Rv genome (NC_000962.3) was used as the reference, to identify genetic variants responsible for INH resistance in the three isolates. Particularly, previously reported resistance genes or loci, including *katG*, *furA-katG*, *mabA-inhA* promoter, *inhA*, *ahpC*, *oxyR-ahpC* intergenic, *iniCAB*, *Rv0340*, *fabD*, *kasA*, *fbpC*, *srmR*, *accD6*, *efpA*, *fadE24*, *ndh*, *Rv1592c*, *Rv1772*, and *nhoA* were selected and analyzed (26). Variants were called when the coverage of a specific position was higher than 50× and variant frequency was >20% as previously described (27). The detailed method for comparative genome analysis was described in Supplemental Materials and Methods.

### Construction of cloning plasmid and transformation

A pOLYG plasmid with a hygromycin resistance marker was introduced into *Escherichia coli* DH5α, and the resulting cells were streaked on hygromycin-containing Luria-Bertani (LB) agar. A single colony was then transferred to LB broth and incubated for 16 hours. The plasmid was isolated using the QIAprep spin miniprep kit (QIAGEN). The entire *furA-katG* operon, including the promoter region, was amplified from the three clinical isolates. The vector backbone and *furA-katG* amplicon were subjected to restriction digestion with *Xba*I and *Hind*III, followed by ligation to produce the cloned plasmids listed in Table S1. The plasmids containing the insert were then electroporated into a catalase-negative INH-resistant *M. tuberculosis* strain GA03 using an Eporator (Eppendorf, Germany) with the setting of 2.5 kV, 25 µF, and resistance at 1,000 Ω. The presence of mutations of interest in the insert was confirmed by Sanger sequencing using additional forward and reverse primers (Table S2) to cover the entire *furA-katG* operon as previously described (28).

### Minimal inhibitory concentration

The minimal inhibitory concentrations (MICs) of INH for *M. tuberculosis* H37Rv, GA03 (INH-resistant), GA03 harboring pOLYG (vector control), and GA03 harboring pOLYG *furA-katG* insert from strains M_11806, D1_12327, and D2_12328 were determined using agar proportion method as previously described (29). All experiments were conducted in triplicate (detailed method is described in Supplemental Materials and Methods).

### Semi‐quantitative catalase assay

The catalase test was conducted for *M. tuberculosis* transformants harboring the *furA-katG* operon from three strains (M_11806, D1_12327, and D2_12328), *M. tuberculosis* H37Rv (positive control), and strain GA03 without the plasmid (negative control). All experiments were conducted in triplicate as described previously (28).

### Modeling of INH binding in wild and mutant katG

The three-dimensional structure of the wild type and mutated katG proteins from three experimental strains was built by protein homology modeling with the crystal structure of the katG protein of *M. tuberculosis* (1SJ2) (30) using the server SWISS-MODEL with default parameters. The model was further analyzed using the Discovery Studio visualizer (Accelrys), and the Ramachandran plot was examined to ensure that the structure of the model was not in any unfavorable region. INH-heme binding model in the constructed was created by superimposing the katG structure with the drug.

Furthermore, the Protein Variation Effect Analyzer (PROVEAN), a web server tool used to calculate pairwise alignment scores between a protein sequence and a large protein database with precomputed scores (31), was utilized to predict the functional consequences of the novel amino acid substitutions on the KatG protein.

## RESULTS

### The clinical case

**Fig 1:**
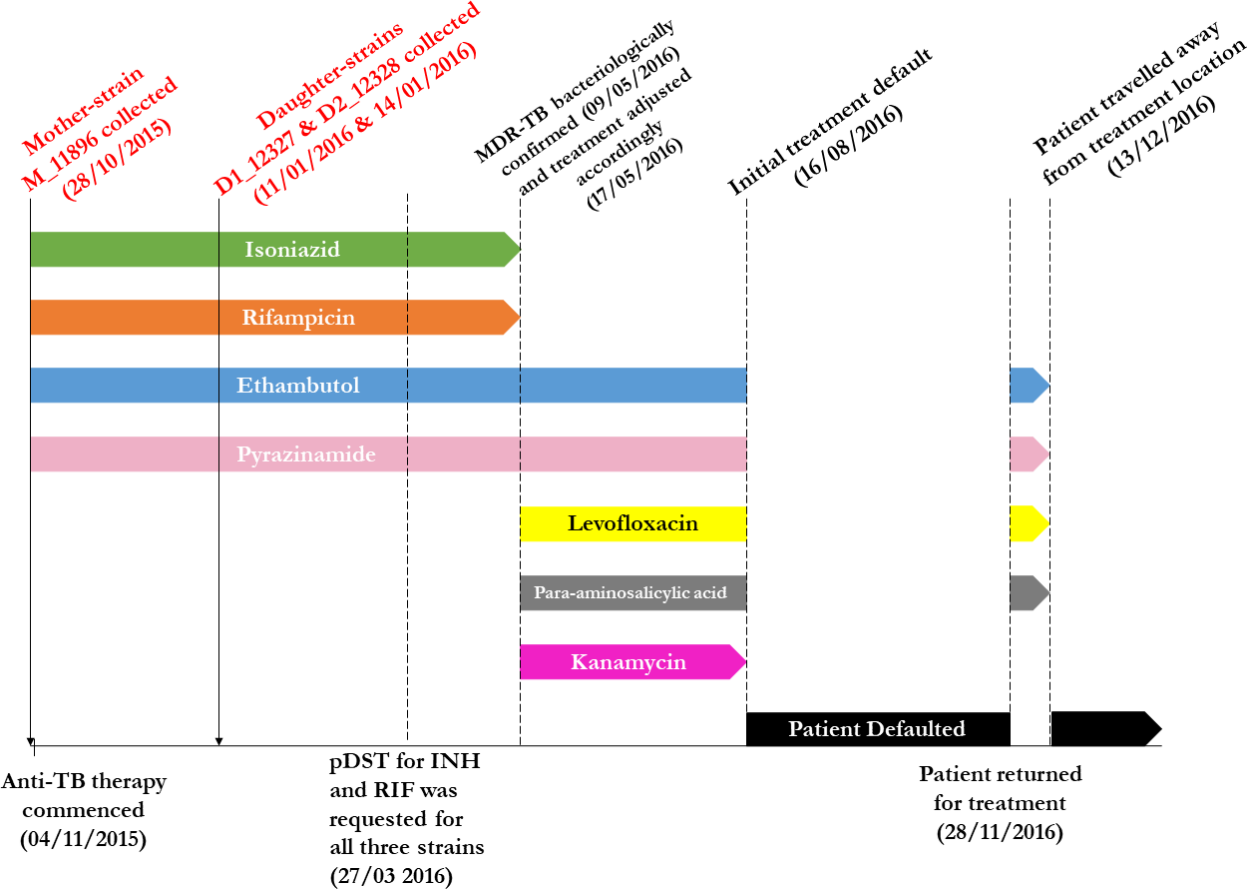
Treatment history of the clinical case. A 60-year-old patient presenting with presumptive symptoms of TB visited a chest clinic for medical consultation on 5 October 2015. A sputum sample was collected for AFB culture. On 28 October 2015, pulmonary TB was confirmed when the first *M. tuberculosis* strain, M_11806 (mother strain), was isolated. Standard anti-TB therapy (2HRZE) was initiated on 4 November 2015. However, despite treatment, the patient’s symptoms persisted. Two follow-up consultations took place on 7 December and 14 December 2015, during which two additional sputum samples were collected. Subsequently, on 11 January and 14 January 2016, two *M. tuberculosis strains*, D1_12327 and D2_12328 (daughter strains), were isolated from these samples. Consequently, the patient missed several medical appointments in February and March 2016. The patient sought medical consultation again on 27 March 2016. Phenotypic DSTs for INH and RIF were requested for all *M. tuberculosis* isolates (including the mother strain and the two daughter strains) collected from this case. On 9 May 2016, MDR-TB was confirmed based on the pDST results. Consequently, RIF and INH were discontinued and replaced with levofloxacin, para-aminosalicylic acid, and kanamycin on 17 May 2016. However, the patient defaulted the treatment in August 2016 due to unknown reasons. He returned to treatment in late November 2016. Disappointedly, he defaulted again 2 weeks later as he emigrated from Hong Kong.

On 5 October 2015, a 60-year-old male patient with presumptive symptoms of TB visited a chest clinic for medical consultation. A sputum sample was collected for acid-fast bacilli (AFB) culture, and on 28 October 2015, pulmonary TB was confirmed when the first *M. tuberculosis* strain, M_11806 (mother strain), was isolated. Standard anti-TB therapy (2HRZE) was initiated on 4 November 2015. However, as the symptoms persisted, two follow-up consultations on 7 December and 14 December 2015, resulted in the collection of two additional sputum samples. On 11 January and 14 January 2016, respectively, two *M. tuberculosis* strains, D1_12327 and D2_12328 (daughter strains), were isolated from these samples. Drug susceptibility tests for RIF and INH were requested for all three strains on 27 March 2016. The results, obtained on 9 May 2016, revealed that the M_11806 strain was susceptible to INH but resistant to RIF. Interestingly, the two daughter strains, D1_12327 and D2_12328, were found to be INH-resistant, resulting in a multidrug-resistant TB (MDR-TB) case. On 17 May 2016, the therapy was adjusted to include levofloxacin, para-aminosalicylic acid, and kanamycin. Unfortunately, the patient defaulted on treatment twice afterward due to travel outside of Hong Kong. The detailed treatment timeline is presented in Fig. 1.

All three strains were retrospectively sent to the University of Hong Kong for repeat pDST using the Bactec MGIT 960 SIRE kit and PZA kit. The results confirmed that the mother strain was susceptible to INH and EMB but resistant to RIF, PZA, and STR. Both daughter strains exhibited conversion to INH resistance at concentrations of 0.1 mg/L and 0.4 mg/L (Table 1).

**TABLE 1:** Phenotypic and genotypic characteristics of *M. tuberculosis* strains

		*M. tuberculosis* strains isolated from the same patient		
		M_11806	D1_12327	D2_12328
Phenotypic drug susceptibility pattern^*a*^				
		INH-S, RIF-R, PZA-R, EMB-S, STR-R	INH-R, RIF-R, PZA-R, EMB-S, STR-R (MDR-TB)	INH-R, RIF-R, PZA-R, EMB-S, STR-R (MDR-TB)
Genotypic drug susceptibility pattern				
Related TB drugs	Resistance genes/loci	Mutations ^*b*^ (Allele frequency)		
Isoniazid (INH)	*katG*	Arg463Leu (100.0%)	Arg463Leu (99.8%) Pro232Pro (33.0%) Pro232Leu (67.0%)	Arg463Leu (99.8%) Pro232Pro (91.0%) Pro232Leu (9.0%) Gln461Stop (97.0%)
	*furA-katG*	WT^*c*^	WT	WT
	*inhA*	WT	WT	WT
	*mabA-inhA*	WT	WT	WT
	*oxyR-ahpC*	WT	WT	WT
	*ahpC*	WT	WT	WT
	*Rv0340*	WT	WT	WT
	*iniA*	Ser501Ser (99.0%)	Ser501Ser (99.0%)	Ser501Ser (99.0%)
	*iniB*	WT	WT	WT
	*iniC*	WT	WT	WT
	*srmR*	WT	WT	WT
	*fabD*	WT	WT	WT
	*kasA*	WT	WT	WT
	*accD6*	Asp200Asp (98.0%) Asp229Gly (98.0%)	Asp200Asp (97.0%) Asp229Gly (98.0%)	Asp200Asp (98.0%) Asp229Gly (98.0%)
	*fbpC*	WT	WT	WT
	*fadE24*	WT	WT	WT
	*efpA*	WT	WT	WT
	*Rv1772*	WT	WT	WT
	*Rv1592c*	Ile322Gly (99.0%)	Ile322Gly (94.0%)	Ile322Gly (95.0%)
	*ndh*	WT	WT	WT
	*nhoA/nat*	WT	WT	WT
Rifampicin (RIF)	*rpoB*	His526Leu (98.0%)	His526Leu (97.0%)	His526Leu (96.0%)
Pyrazinamide (PZA)	*pncA*	−11 A > C (98.0%)	−11 A > C (93.0%)	−11 A > C (98.0%)
	*rpsA*	WT	WT	WT
Ethambutol (EMB)	*embB*	WT	WT	WT
Streptomycin (STR)	*rpsL*	WT	WT	WT
	*rrs*	WT	WT	WT

^*a*^ Phenotypic drug susceptibility pattern was determined by Bactec MGIT 960 SIRE kit and PZA kit.

^*b*^ Variants are called based on H37Rv reference genome NC_000962.3.

^*c*^ WT: Wildtype.

### Clonality of the three *M. tuberculosis* strains

**Fig 2:**
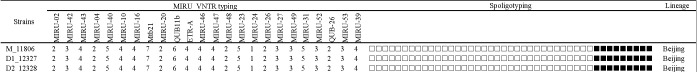
24-loci MIRU-VNTR and spoligotyping of the three *M. tuberculosis* strains from the same patient.

**Fig 3:**
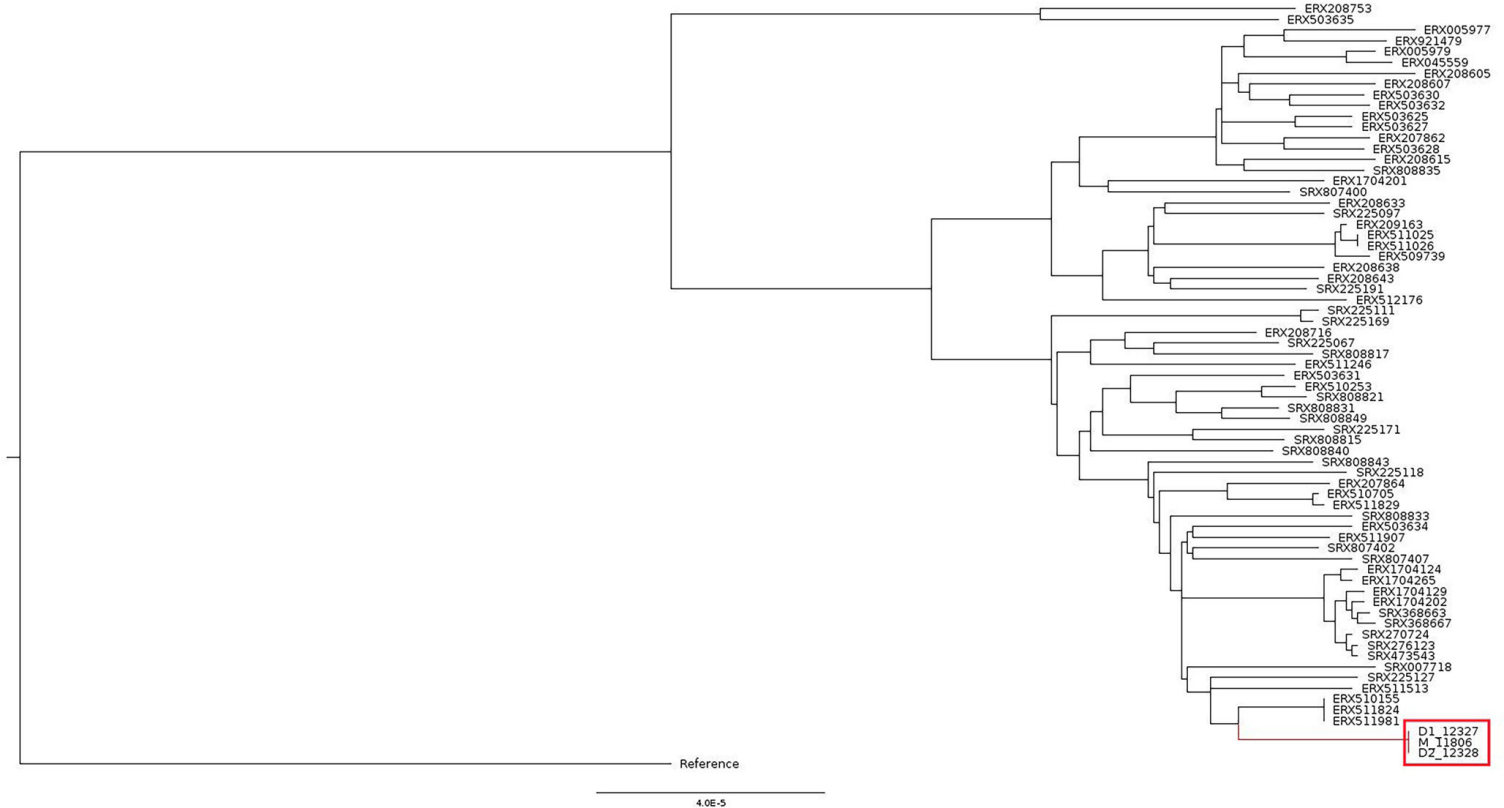
Core-genome SNP phylogeny of the three *M. tuberculosis* strains in this study. Strains M_11806, D1_12327, and D2_12328 (indicated inside the red grid) had identical core genomes (i.e., no core genome SNP difference), suggesting that they belong to the same clone. Regarding the phylogenetic placement, these strains were clustered with other *M. tuberculosis* strains of lineage 2.2.2., which were also classified as Beijing/Asia Ancestral 1 sub-lineage.

The 24-loci MIRU-VNTR analysis and the *in silico* spoligotyping revealed that M_11806, D1_12327, and D2_12328 shared the same genetic patterns, suggesting that they originated from the same clonal population and were classified as belonging to the Beijing lineage (Fig. 2). The clonality of the three strains was validated by core-genome SNP phylogeny, which showed that they shared 100% identical core genome, which refers to the genome excluding the accessory genes like drug resistance genes and virulence genes (genomes highlighted in red grid in Fig. 3). In addition, the phylogenetic placement in the tree demonstrated that the three isolates were clustered with *M. tuberculosis* strains of lineage (L) 2.2.2., supporting that these isolates belong to Beijing/Asia Ancestral 1 sub-lineage.

### Comparative genome analysis and INH resistance-conferring mutations

Each isolate was sequenced and yielded nearly 6 million sequence reads with an average read length of 147.3 bp and a depth of coverage of 186.4× (Table S3). The reads provided uniform coverage across the entire reference genome NC_000962.3. After removing polymorphisms in the *pe/ppe* gene families and large deletions, which are markers of the Beijing genotype, and fixing the allelic frequency to ≥20×, we discovered a total of 1,621 mutations shared by the three isolates when compared to *M. tuberculosis* H37Rv. A total of 4 and 16 unique single nucleotide polymorphisms were found only in isolates D1_12327 and D2_12328, respectively (Fig. S1). A comprehensive comparative analysis of WGS was performed to detect INH resistance-conferring mutations in 19 structural and 2 regulatory regions (Table 1). Two novel mutations in *katG* were discovered in on-treatment strains, Pro232Leu substitution (CCG→CTG, chromosomal position 2155417) at an allele frequency of 67% and Gln461Stop termination (CCG→CTG, chromosomal position 2155417) at an allele frequency of 97% in the D1_12327 and D2_12328 strains, respectively (Table 1). Notably, Pro232Leu substitution was also detected in the D2_12328 strain with an allele frequency of 9.0% although Sanger sequencing confirmed only the Gln461Stop mutation in this strain.

Moreover, all three strains exhibited mutations that were consistent with pDST. Specifically, a missense mutation, His526Leu, was identified in *rpoB*, which confers resistance to RIF. Additionally, a nucleotide change, −11A > C, was found in the promoter region of *pncA*, which confers resistance to PZA (Table 1).

### Functional validation of novel katG mutations

The entire *furA-katG* operon, along with the upstream promoter region, from M_11806, D1_12327, and D2_12328 strains were successfully transformed into the catalase-negative strain, GA03 (Fig. S2). The MICs of INH were determined for *M. tuberculosis* H37Rv, strain GA03, GA03 containing the vector alone and the vector cloned with the *furA-katG* operon from M_11806, D1_12327, and D2_12328 strains, respectively.

**Fig 4:**
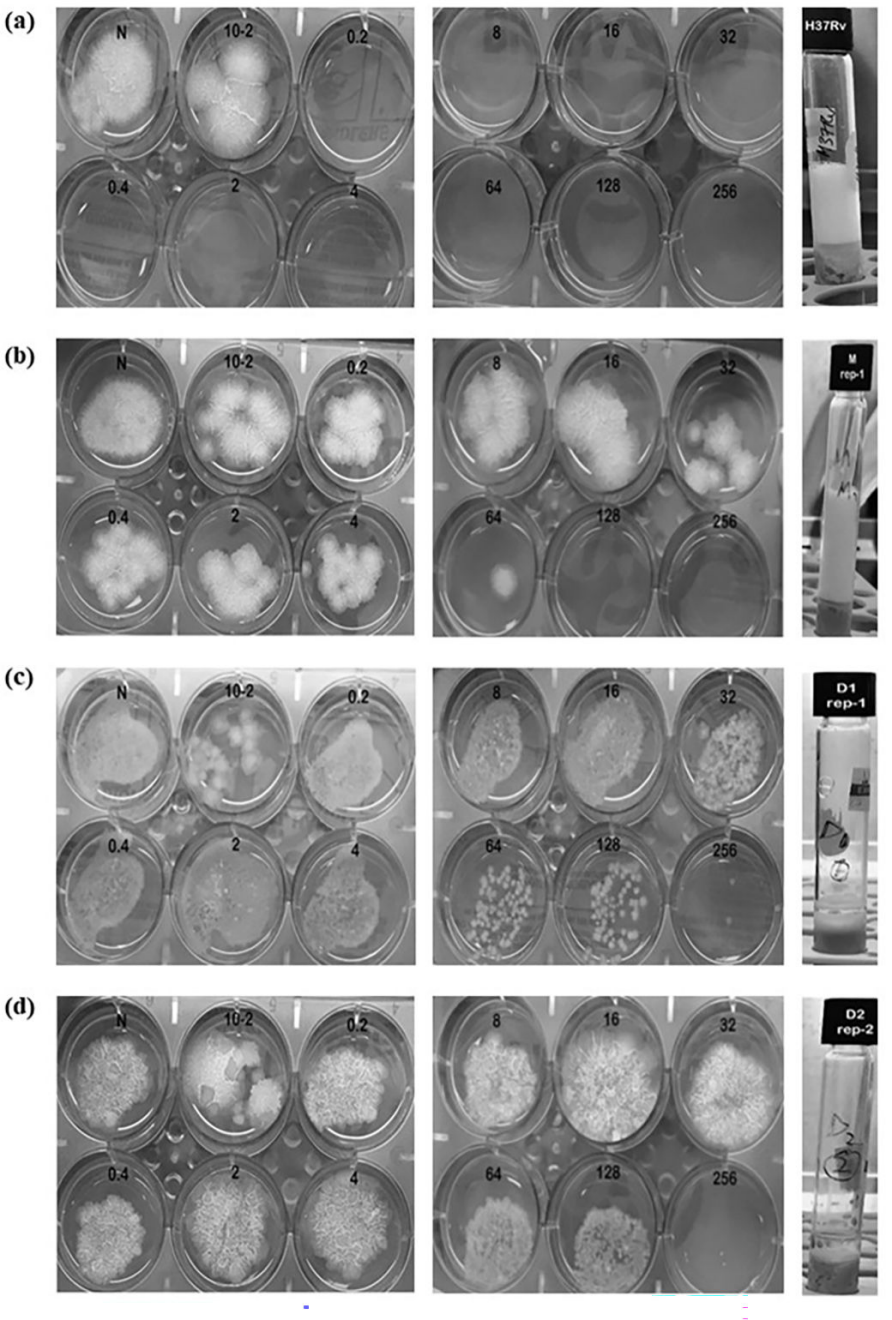
MIC and catalase activity using the agar proportion method and semi-quantitative catalase activity of the *katG* transformants and H37Rv. The label on the plate indicates the serial dilution of INH in mg/L; N denoted drug-free well inoculated with neat bacterial concentration, i.e., 10,000 CFU/well; 10–2 denoted drug-free well inoculated with 1:99 diluted bacteria, i.e., 100 CFU/well. (**a**) H37Rv reference strain (wild; control) with MIC = 0.2 mg/L, catalase = 30 mm; (**b**)GA03 (pOLYG::M_11806) with MIC = 32 mg/L, catalase = 25 mm; (**c**) GA03 (pOLYG::D1_12327) with MIC = 256 mg/m, catalase = 3 mm; and (**d**) GA03 (pOLYG::D2_12328) with MIC = 256 mg/L, catalase = 1 mm.

Strain GA03 carried a nonsense mutation, Q434Stop, in *katG*, which resulted in the complete loss of catalase activity and high INH MICs of 256 mg/L. As depicted in Fig. 4 and Table 2, the introduction of the *furA-katG* operon of M_11806 successfully reduced the MIC to 32 mg/L, partially restoring susceptibility to INH. However, the transformants carrying the constructs pOLYG::*katG* Q461Stop and pOLYG::*katG* P232L remained highly resistant to INH, with MICs of 128 mg/L and 256 mg/L, respectively. We also evaluated the impact of these mutations by measuring the functional activity of catalase produced by the transformants carrying the constructs with novel *katG* mutations. The results showed that the complementation of the *furA-katG* operon of M_11806 had recovered the catalase activity of GA03. However, constructs with novel mutations substantially lost catalase activity, with a bubble height of ≤3 mm, which was ≥eightfold lower than the construct carrying pOLYG::M_11806 and *M. tuberculosis* H37Rv. Remarkably, catalase production was lower for the construct that had a termination mutation than for the one with the amino acid substitution.

**TABLE 2:** The minimal inhibitory concentration results of transformed mutants and reference strains

Strain	Characteristics	MIC (mg/L)	Catalase activity
GA03	A clinical isolate of *M. tuberculosis* with nonsense mutation, Q434Stop, in *katG*, resulting in negative catalase and high-level INH resistance	256	0
GA03 (pOLYG)	*M. tuberculosis* GA03 that was transformed with intact mycobacterial expression vector, pOLYG	256	0
GA03 (pOLYG::M_11806)	*M. tuberculosis* GA03 that was transformed with pOLYG carrying entire *furA-katG* operon from M_11806, pOLYG::Wild katG	32	25
GA03 (pOLYG::D1_12327)	*M. tuberculosis* GA03 that was transformed with pOLYG carrying entire *furA-katG* operon from D1_12327, pOLYG::*katG* P232L (CCG→CTG)	256	3
GA03 (pOLYG::D2_12328)	*M. tuberculosis* GA03 that was transformed with pOLYG carrying entire *furA-katG* operon from D2_12328, pOLYG::*katG* Q461Stop (CAG→TAG)	256	1
H37Rv	Reference strain used in MIC test	0.2	30

### Protein model for prediction of the effect of novel mutations on INH binding

**Fig 5:**
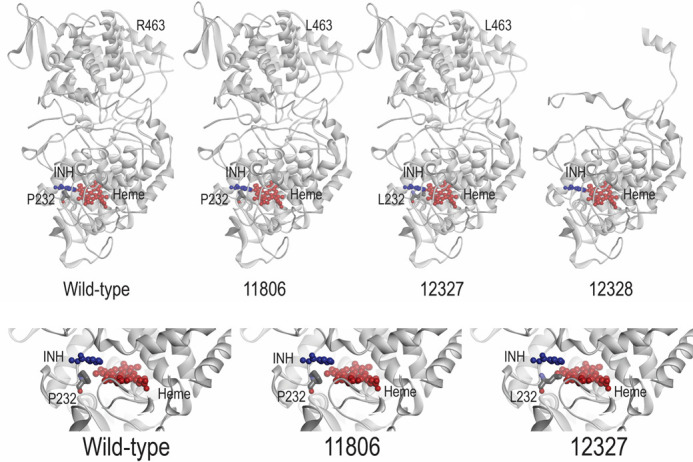
KatG protein model of H37Rv (wildtype) and *M. tuberculosis* strains M_11806, D1_12327, and D3_12328 interacted with INH and heme.

To explain the impact of the mutations on the binding with INH, we modeled a three-dimensional (3D) structure of katG (mutant and wild) with INH and heme (Fig. 5). The natural polymorphism, R463L, in all three isolates is located far away from the active binding site and does not alter any structural properties of the protein. However, the other two mutations (Pro232Leu and Gln461Stop) in D1_12327 and D2_12328, respectively, have a significant effect on the catalytic activity of INH. The proline residue in wild-type katG at codon 232 is crucial for the binding of INH/Heme, as previously reported (32). The substitution of proline with leucine in D1_12327 (Pro232Leu) interfered with the binding of INH to the enzyme in the active site, limiting the access of INH to the iron atom, where catalysis takes place. The mutation in D2_12328 (Gln461Stop) results in a truncated protein from 461 residues to 741 residues in the C-terminus (280 total residues), causing the loss of enzymatic activity, as the protein cannot fold correctly. The deleterious impact of these two novel mutations on the catalase-peroxidase protein was further evaluated using PROVEAN (31). The PROVEAN scores of −9.818 and −10.943 were obtained for Pro232Leu and Gln461Stop, respectively, which are considered deleterious to the function of the catalase-peroxidase enzyme.

## DISCUSSION

INH remains the backbone of the standard treatment regimen for TB. Resistance to INH is often associated with a variety of polymorphisms, including complete loss of *katG* (26), single amino acid substitutions, termination mutations (33), deletions, and frameshift insertions in *katG* (34). These mutations can have varying effects on *katG* enzymatic activity and the level of resistance to INH (35).

In this study, we documented the co-emergence of MDR-TB clones in a patient during the standard course of treatment and demonstrated the causal role of emerging *katG* mutations in the development of INH resistance. The patient was initially infected with a strain of *M. tuberculosis* that was resistant to RIF and PZA but susceptible to INH and EMB. Without the guidance of pDST, the standard short-course 6-month regimen was administered to the patient. However, no clinical improvement was observed during the treatment. After 2 months of treatment, two consecutive *M. tuberculosis* strains were collected, both of which were found to be INH-resistant. Standard 24-MIRU-VNTR and core-genome phylogeny confirmed that the strains were clonally related and belonged to the Beijing lineage. Minimal genetic variations were observed across the whole genome of the three isolates (<15 SNPs identified in accessory genes at ≥20% allelic frequency, and no SNP difference identified in the core genome), supporting the clonal relatedness of the strains. These findings suggest that the two INH-resistant strains evolved from the pre-treatment INH-susceptible strain during treatment.

Comparative WGS analysis of the entire *furA-katG* and *mabA-inhA* genetic regions as well as previously reported genetic stretches associated with INH resistance was performed (26). We found the absence of INH resistance-conferring genetic polymorphisms in all loci except for two novel mutations in *katG*, P232L, and Q461Stop, in the strains isolated during the treatment. *M. tuberculosis* has a natural propensity of spontaneous mutations that confer INH resistance in approximately 1 out of 10^6^–10^8^ bacterial populations (9). Suboptimal drug pressure, either due to patient-related factors or programmatic mismanagement, could facilitate the selection of mutant (drug-resistant) clones. The *M. tuberculosis* lineage has also been shown to affect the speed of acquisition of genetic variants. The Beijing lineage, in particular, is hypermutable compared to other lineages (36), which may have increased the chance of the emergence of INH resistance while the patient was undergoing standard treatment.

The emergence of drug-resistant clones in a single patient has caught substantial attention in the last several decades. While previous studies have produced conflicting results regarding the speed of emergence and fixation of drug-resistant clones *in vivo*, recent research utilizing highly sensitive next-generation sequencing technologies has shown that there is a high level of diversity of mycobacterial strains within individual patients (37, 38). Studies that have examined serial sputum samples from single or multiple patients under standard treatment regimens have demonstrated the emergence and coexistence of multiple drug-resistant-conferring alleles. The ultimate fixation of these alleles is determined by their fitness cost and level of resistance. Generally, mutations that confer high levels of resistance but have low fitness costs are fixed in patients under therapy and subsequently transmitted. For example, Sun et al. studied three serial sputum samples collected from a single patient and identified four INH-resistant associated mutations (three in *katG* and one in *inhA*). After 23 months, only one variant with a high resistance level and low fitness cost was fixed (4). A similar study examining RIF and STR-associated mutations in sputum collected from the same patient found that high drug resistance-conferring and low fitness cost genetic mutations were fixed (39, 40). Furthermore, previous studies have revealed that *M. tuberculosis* employs various compensatory mechanisms to counteract the loss of catalase activity caused by mutations in *katG*. These mechanisms include the upregulation of alkylhydroperoxidase (AhpC) expression, which has been shown to restore catalase activity in the presence of *katG* mutations (41, 42). Sherman et al. demonstrated that ahpC and katG share reactive nitrogen intermediates and organic peroxides, which contribute to enhancing the compensatory mechanism when mycobacteria experience a loss of catalase function due to katG mutations (43). However, it is important to note that only a small proportion (~9%) of *katG* mutants were found to carry mutations in the *oxyR-ahpC* region, resulting in the overexpression of AhpC (44). Similarly, in our study, no variants were identified in the *oxyR-ahpC* region in both daughter strains. Therefore, it is speculated that both strains may compensate for the reduced catalase activity through epigenetic mechanisms (45).

In this study, although we did not obtain data supporting the role of two novel mutations in relation to the fitness cost, we demonstrated that both mutations confer high INH resistance through functional genetics. To determine the impact of the mutations, a katG-deleted *M. tuberculosis* strain was transformed with a mycobacterial vector (pOLYG) carrying the *furA-katG* operon from the two strains (Table 2). The introduction of mutants *katG* P232L and Q461Stop resulted in high INH resistance with MICs of 256 mg/L and 128 mg/L, respectively, indicating that *katG* genes with these mutations are defective. These MIC results are consistent with a range of 0.19 to 256 mg/L obtained for single missense mutations of *katG* in clinical isolates by Ramaswamy et al. (26). However, it is worth noting that Ramaswamy et al. (26) did not conduct a transformation experiment to confirm the causal role of the identified substitutions. Similarly, Kandler et al. (34) and Torres et al. (6) also identified and cloned several novel mutations in *katG* that increased the MIC of the transformants. Nonetheless, the MIC results in our study were higher than those reported in these two studies, which could be due to the difference in the identified mutations and the models (H37Rv and *Mycobacterium smegmatis*) used for the functional assay in the respective studies.

Catalase-peroxidase, encoded by *katG*, plays a crucial role in converting pro-INH to an active form that has pleiotropic effects on the bacilli (46). Mutations in the coding stretch of *katG* may reduce the catalytic activity of the gene product, potentially leading to decreased drug susceptibility (26). *In vitro* evaluation of the catalase activity of the transformants can enable the assessment of the impact of novel mutations in *katG* (26). As determined by catalase activity, our study also revealed that the *M. tuberculosis* strain, GA03, which harbored mutated *katG* (P232L and Q461Stop), has reduced catalase activity by more than eightfold compared to the transformants carrying the gene from the pre-treatment strain, suggesting that the mutations resulted in a defective protein. Previous studies have shown that mutants associated with high MIC were characterized by a drastic reduction in catalase activities (35, 47).

To consolidate the role of the two novel mutations in INH resistance and understand the effect of amino acid substitution and termination, 3D protein-INH binding models were constructed. The P232L substitution is located very close to the INH binding site and could potentially obstruct the binding of INH. As expected, the crystallographic analysis showed that the P232L substitution reduces INH-*KatG* binding affinity, affecting the conversion of the prodrug to the active form, which is a critical step in the mechanism of action of INH. Transformants carrying this mutation were associated with high resistance to INH, as demonstrated by the MIC study. On the other hand, the termination mutation (Q461Stop) generates a truncated protein at 461 residues without 280 residues in the C-terminal domain. The Q461Stop mutation truncates gene transcription, limiting the activity of the enzyme and causing high-level INH resistance.

In conclusion, our results showed that the two *katG* mutations, P232L and Q461Stop, contributed to the co-emergence of INH-resistant clones during the standard course of anti-TB therapy. We confirmed the presence of two independent mutations that evolved due to anti-TB stress from a parental strain and experimentally demonstrated the functional role of both mutations in conferring INH resistance. The inclusion of these mutations in the design of molecular assays can improve diagnostic performance.

## Data Availability

The genomes of the three M. tuberculosis strains were submitted to the NCBI GenBank and were assigned the accession numbers SAMN34123194, SAMN34123195, and SAMN34123196, respectively.

## SUPPLEMENTAL MATERIAL

Supplemental material spectrum.02133-23-s0001.pdf Supplemental methods, supplemental tables, and Supplemental figures.

ASM does not own the copyrights to Supplemental Material that may be linked to, or accessed through, an article. The authors have granted ASM a non-exclusive, world-wide license to publish the Supplemental Material files. Please contact the corresponding author directly for reuse.
