# Supplementary material for: Genetic mechanisms of co-emergence of INH-resistant Mycobacterium tuberculosis strains during the standard course of antituberculosis therapy
Source: Microbiol Spectr. 2024 Mar 11;12(4):e02133-23. doi: 10.1128/spectrum.02133-23 (PMC10986572; doi:10.1128/spectrum.02133-23)
Supplement: Supplemental material — Supplemental methods, supplemental tables, and Supplemental figures. [file spectrum.02133-23-s0001.pdf]

Supplemental Material

Genetic Mechanisms of Stepwise Development of INH Resistance in *Mycobacterium tuberculosis* Strains during the Standard Course of Antituberculosis Therapy

Contents

ONLINE SUPPLEMENTARY METHODS 2
Comparative Genome Analysis. 2
Core-genome phylogeny for confirmation of clonality 2
Minimal Inhibitory Concentration (MIC). 4
ONLINE SUPPLEMENTARY TABLES 5
Table S1: Bacterial isolates and cloned plasmids generated in this study 5
Table S2: PCR and sequencing primers used in cloning experiment of this study 6
Table S3: The summary of the quality of sequencing reads and alignment 7
Table S4: Sequencing reads from previous studies included in the core-genome phylogeny 8
ONLINE SUPPLEMENTARY FIGURES 12
Figure S1. .... **Error! Bookmark not defined.**
Figure S2. 13
REFERENCES 14

ONLINE SUPPLEMENTARY METHODS

Comparative Genome Analysis.

Quality assessment of MiSeq sequenced data (FASTQ format) was conducted using fastp v0.23.2[1]. Burrows-Wheeler Aligner- Maximal Exact Matches 2 (*BWA-MEM2*)[2] was used to map reads to the *M. tuberculosis* H37Rv (GenBank NC_000962.3, taxid:83332). SAMtools, mpileup, and VCFtools were used to carry out the downstream analysis and variant calling. Polymorphisms including large InDels and variants in *pe/ppe* genes families and mobile genetic elements were excluded for variant calling to avoid inaccuracy of the identification of mutation.

Core-genome phylogeny for confirmation of clonality

To construct the core-genome single nucleotide polymorphism (SNP) phylogeny, the GenBank file of the *M. tuberculosis* reference strain H37Rv was first downloaded from the NCBI RefSeq database using ncbi-genome-download v0.3.1 (*Kblin. KBLIN/NCBI-genome-download: Scripts to download genomes from the NCBI FTP servers [Internet]. GitHub. [cited 2023Apr11]. Available from: https://github.com/kblin/ncbi-genome-download*). The raw sequencing reads of other L2 strains from previous studies were downloaded from the Sequence Read Archive (SRA) or European Nucleotide Archive (ENA) using fastq-dl (*rpetit3. RPETIT3/FASTQ-DL: DOWNLOAD FASTQ files from SRA or ENA repositories. [Internet]. GitHub. [cited 2023Apr11]. Available from: https://github.com/rpetit3/fastq-dl*).

All reads were analyzed using Bactopia v2.2.0 - a pipeline for complete analysis of bacterial genomes[3]. In brief, SNPs were first called against the reference genome H37Rv using Snippy v4.6.0 (*Tseemann. Tseemann/snippy: Rapid haploid variant calling and core genome alignment*

[Internet]. GitHub. [cited 2023Apr11]. Available from: https://github.com/tseemann/snippy). Core-genome SNPs (cgSNPs) calling, and alignment were performed using snippy-core v4.6.0 (*Tseemann. Tseemann/snippy: Rapid haploid variant calling and core genome alignment [Internet]. GitHub. [cited 2023Apr11]. Available from: https://github.com/tseemann/snippy*). The core-genome SNP phylogeny was reconstructed using the substitution model GTR+Γ with IQ-TREE v2.2.0[4]. The reconstructed phylogeny was visualized using figtree v1.4.4 (*Figtree. [cited 2023Apr11]. Available from: http://tree.bio.ed.ac.uk/software/figtree/*). The pairwise SNP distance was calculated using snp-dists v0.8.2 (*Tseemann. Tseemann/SNP-Dists: Pairwise SNP distance matrix from a Fasta sequence alignment [Internet]. GitHub. [cited 2023Apr11]. Available from: https://github.com/tseemann/snp-dists*).

Minimal Inhibitory Concentration (MIC)

The bacterial colonies of transformants harboring mutations of interest and control strains were inoculated aseptically into 10ml Middlebrook 7H9 broth supplemented with 10% OADC and 0.1% Tween80. The inoculum was incubated at 37°C for about 15 days until an optical density of 1.0 McFarland was achieved. The bacterial suspension was vortexed with sterile beads and allowed to stand for 15-20 minutes to settle large clumps. The supernatant was then transferred to a sterile tube. The bacterial suspension was diluted to yield a 0.5 McFarland (equivalent to 1 X 10^7 CFU/ml), which was further diluted to 10^2 and 10^4-fold to obtain standard (equivalent to 1 X 10^5 CFU/ml) and control (equivalent to 1X10^3 CFU/ml) inocula. Subsequently, 100µl of the aliquot from the respective inoculum was transferred to 6-well plates containing Middlebrook 7H10 agar supplemented with INH in concentrations ranging from 0.01µg/ml to 256 µg/ml. All experiments were conducted in triplicate, and the MIC was interpreted as the minimum concentration of INH that inhibits 99% of *M. tuberculosis* compared to drug-free wells.

ONLINE SUPPLEMENTARY TABLES

Table S1: Bacterial isolates and cloned plasmids generated in this study

Sr no	Bacterial strain/s and plasmid	Description	Source/reference
1	*E. coli* DH5α with and without plasmid	huA2 lac *(del)*U169 phoA glnV44 *ϕ80′* lacZ*(del)M15 gyrA*96 recA1 relA1 endA1 thi-1 hsdR17	5
2	pOLYG plasmid	*E. Coli-Mycobacterium tuberculosis* shuttle vector, nonexpressive: P^{AL5000} origin of replication, Contain hygromycin resistance marker	5
3	pOLYG::M_11806	pOLYG containing entire *furA-katG*, from *M.tuberculosis* (11806) contain a mutation at *katG*4 R63L(CGG>CTG)	This study
4	pOLYG::D1_12327	pOLYG containing entire *furA-katG*, from *M.tuberculosis* (12327) contain mutation at *katG* R463L (CGG→CTG) and codon P232L (CCG→CTG)	This study
5	pOLYG::D2_12328	pOLYG containing entire *furA-katG*, from *M.tuberculosis* (12328) contain a mutation at *katG* R463L (CGG→CTG) and codon Q461Stop (CAG→TAG)	This study

Table S2: PCR and sequencing primers used in cloning experiment of this study

Primer name	Direction	Sequence	Amplicon size (bp)
KatG-cloning-1F	F	TATAATCTAGAGTGCCCGAGCAACACCCAC	2,421
KatG-cloning-R	R	TATAAAAGCTTATCGCACATCCAGCACATTTCG	
KatG-cloning-2F	F	TATAATCTAGACTACTGGGGTCTATGTCCTGATTG	2,517
KatG-cloning-R	R	TATAAAAGCTTATCGCACATCCAGCACATTTCG	
KatG-cloning-3F	F	TATAATCTAGATTGTCTAGTGTGTCCTCTATACCGGAC	2,911
KatG-cloning-R	R	TATAAAAGCTTATCGCACATCCAGCACATTTCG	
KatG-cloning-4F	F	TATAATCTAGACATCGGAACATACGAAGGCTG	3,037
KatG-cloning-R	R	TATAAAAGCTTATCGCACATCCAGCACATTTCG	
KatG-cloning-5F	F	TATAATCTAGACTCCAGCTGCCACCGGGAGC	3,790
KatG-cloning-R	R	TATAAAAGCTTATCGCACATCCAGCACATTTCG	
pOLYG-seqF	F	GCGCAACGCAATTAATGTGAGTTAGC	340 (for intact plasmid)
pOLYG-seqR	R	TTTGCTTGTCTCCGAATCCAACTGG	3,360 (with *furA*-*katG* insert)

Table S3: The summary of the quality of sequencing reads and alignment

Character	Isolates ID		
	**M_11806**	**D1_12327**	**D2_12328**
Number of reads	5,849,450	5,867,562	5,753,440
Average read length	148	146	148
GC%	65.8	65.7	65.9
Percentage of bases with Phredscore ≥ 30	93.8	93.2	93.3
Mapped reads (%)[1]	5,786,701 (98.93%)	5815324 (99.11%)	5695961 (99.00%)
Mean Coverage	188.4X	187.76X	183.1X
Mean mapping quality (%)	59	58.9	58.9

[1] The sequencing reads were mapped to H37Rv reference genome NC_000962.3 with BWA-MEM2

Table S4: Sequencing reads from previous studies included in the core-genome phylogeny

Experiment accession number	Run accession number	Lineage	Reference
ERX208605	ERR234100	L2.2.7	6
ERX208607	ERR234102	L2.2.7	6
ERX503625	ERR538422	L2.2.6	7
ERX503627	ERR538424	L2.2.6	7
ERX503628	ERR538425	L2.2.5	7
ERX503630	ERR538427	L2.2.8	7
ERX503631	ERR538428	L2.2.2	7
ERX503632	ERR538429	L2.2.8	7
ERX503634	ERR538431	L2.2.2	7
ERX503635	ERR538432	L2.1	7
ERX208615	ERR234110	L2.2.4	6
ERX208633	ERR234128	L2.2.1	6
ERX208638	ERR234133	L2.2.1	6
ERX208643	ERR234138	L2.2.1	6
ERX005979	ERR015614	L2.2.9	6
ERX005977	ERR015616	L2.2.10	6
SRX007718	SRR022872	L2.2.2	8
ERX207862	ERR233357	L2.2.5	6
ERX207864	ERR233359	L2.2.2	6
ERX921479	ERR841392	L2.2.10	7

ERX208716	ERR234211	L2.2.2	6
ERX208753	ERR234248	L2.1	6
ERX1704129	ERR1633796	L2.2.2	9
ERX1704124	ERR1633791	L2.2.2	9
ERX1704201	ERR1633868	L2.2.3	9
ERX1704202	ERR1633869	L2.2.2	9
ERX1704265	ERR1633932	L2.2.2	9
SRX807400	SRR1710073	L2.2.3	10
SRX807402	SRR1710074	L2.2.2	10
SRX807407	SRR1710076	L2.2.2	10
SRX808815	SRR1710077	L2.2.2	10
SRX808817	SRR1710079	L2.2.2	10
SRX808821	SRR1710083	L2.2.2	10
SRX808831	SRR1710093	L2.2.2	10
SRX808833	SRR1710095	L2.2.2	10
SRX808835	SRR1710097	L2.2.4	10
SRX808840	SRR1710102	L2.2.2	10
SRX808843	SRR1710105	L2.2.2	10
SRX808849	SRR1710111	L2.2.2	10
SRX225067	SRR671753	L2.2.2	11
SRX225097	SRR671783	L2.2.1	11
SRX225111	SRR671797	L2.2.2	11

SRX225118	SRR671804	L2.2.2	11
SRX225127	SRR671813	L2.2.2	11
SRX225169	SRR671855	L2.2.2	11
SRX225171	SRR671857	L2.2.2	11
SRX225191	SRR671877	L2.2.1	11
ERX045559	ERR067696	L2.2.9	12
ERX209163	ERR234658	L2.2.1	12
ERX509739	ERR550670	L2.2.1	13
ERX510155	ERR551086	L2.2.2	13
ERX510253	ERR551184	L2.2.2	13
ERX510705	ERR551636	L2.2.2	13
ERX511025	ERR551956	L2.2.1	13
ERX511026	ERR551957	L2.2.1	13
ERX511246	ERR552177	L2.2.2	13
ERX511513	ERR552444	L2.2.2	13
ERX511824	ERR552755	L2.2.2	13
ERX511829	ERR552760	L2.2.2	13
ERX511907	ERR552838	L2.2.2	13
ERX511981	ERR552912	L2.2.2	13
ERX512176	ERR553107	L2.2.1	13
SRX368663	SRR1019148	L2.2.2	14
SRX368667	SRR1019152	L2.2.2	14
SRX473543	SRR1173056	L2.2.2	14

SRX270724	SRR833027	L2.2.2	14
SRX276123	SRR847799	L2.2.2	14

ONLINE SUPPLEMENTARY FIGURES

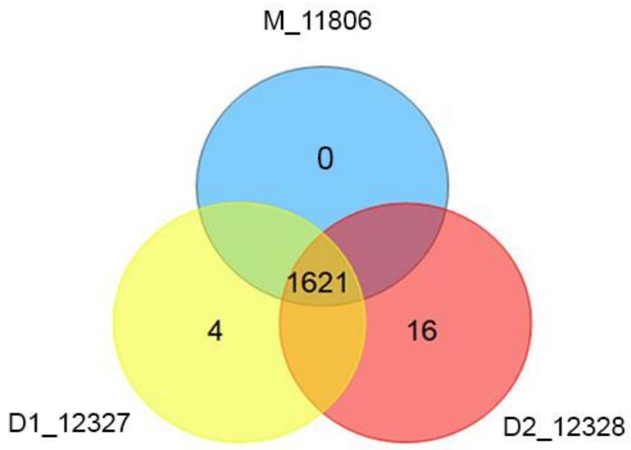

Figure S1: Venn diagram showing variants identified in accessory genes of the three isolates at ≥20% allele frequency

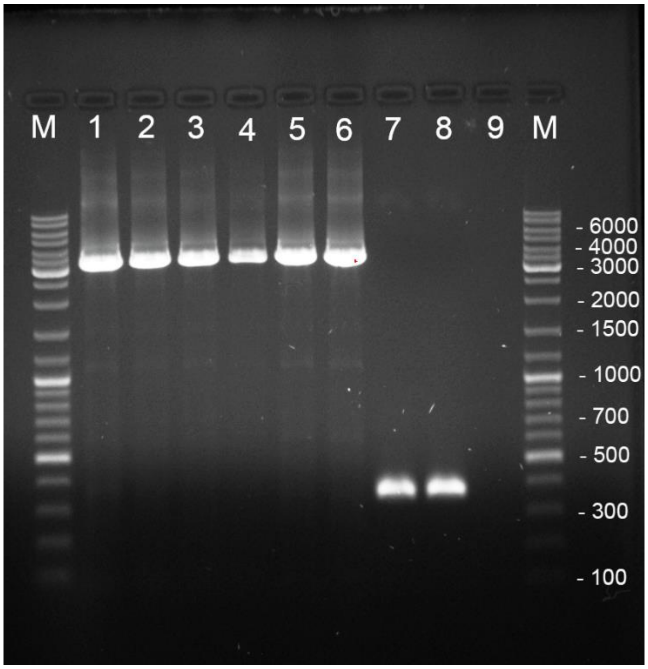

Figure S2: A gel photo demonstrating the amplicons of colony PCR from the *M. tuberculosis* GA03 transformant, confirming the successful cloning of the entire *katG-furA* amplicons into the pOLYG plasmid. The expected amplicon size for intact pOLYG plasmid is 340 bp, whereas successful cloning of entire *furA-katG* operon results in an amplicon of 3,366 bp [*furA-KatG* operon = 3,037 bp + pOLYG plasmid regions flanking the insert = 329 bp]

M: GeneRuler DNA Ladder Mix, Lane 1 and 2: *furA-katG* from M_11806, Lane 3 and 4: *furA-katG* from D1_12327, Lane 5 and 6: *furA-katG* from D2_12328, Lane 7 and 8: Intact pOLYG without insert, and Lane 9: Nuclease Free Water (Negative control).

REFERENCES

1. Chen S, Zhou Y, Chen Y et al. fastp: an ultra-fast all-in-one FASTQ preprocessor. *Bioinformatics* 2018; **34**: i884-i90.
2. Langarita R, Armejach A, Ibanez P et al. Porting and Optimizing BWA-MEM2 Using the Fujitsu A64FX Processor. *IEEE/ACM Trans Comput Biol Bioinform* 2023; **PP**.
3. Petit RA, 3rd, Read TD. Bactopia: a Flexible Pipeline for Complete Analysis of Bacterial Genomes. *mSystems* 2020; **5**.
4. Minh BQ, Schmidt HA, Chernomor O et al. IQ-TREE 2: New Models and Efficient Methods for Phylogenetic Inference in the Genomic Era. *Mol Biol Evol* 2020; **37**: 1530-4.
5. Siu GKH, Yam WC, Zhang Y et al. An Upstream Truncation of the <i>furA-katG</i> Operon Confers High-Level Isoniazid Resistance in a Mycobacterium tuberculosis Clinical Isolate with No Known Resistance-Associated Mutations. *Antimicrobial Agents and Chemotherapy* 2014; **58**: 6093-100.
6. Comas I, Coscolla M, Luo T et al. Out-of-Africa migration and Neolithic coexpansion of Mycobacterium tuberculosis with modern humans. *Nat Genet* 2013; **45**: 1176-82.
7. Kato-Maeda M, Shanley CA, Ackart D et al. Beijing sublineages of Mycobacterium tuberculosis differ in pathogenicity in the guinea pig. *Clin Vaccine Immunol* 2012; **19**: 1227-37.
8. Comas I, Chakravartti J, Small PM et al. Human T cell epitopes of Mycobacterium tuberculosis are evolutionarily hyperconserved. *Nat Genet* 2010; **42**: 498-503.
9. Koch AS, Brites D, Stucki D et al. The Influence of HIV on the Evolution of Mycobacterium tuberculosis. *Mol Biol Evol* 2017; **34**: 1654-68.
10. Luo T, Comas I, Luo D et al. Southern East Asian origin and coexpansion of Mycobacterium tuberculosis Beijing family with Han Chinese. *Proc Natl Acad Sci U S A* 2015; **112**: 8136-41.
11. Zhang H, Li D, Zhao L et al. Genome sequencing of 161 Mycobacterium tuberculosis isolates from China identifies genes and intergenic regions associated with drug resistance. *Nat Genet* 2013; **45**: 1255-60.
12. Casali N, Nikolayevskyy V, Balabanova Y et al. Evolution and transmission of drug-resistant tuberculosis in a Russian population. *Nat Genet* 2014; **46**: 279-86.
13. Merker M, Blin C, Mona S et al. Evolutionary history and global spread of the Mycobacterium tuberculosis Beijing lineage. *Nat Genet* 2015; **47**: 242-9.
14. TB-arc project (tuberculosis antibiotic resistance catalog project). https://www.broadinstitute.org/genome-sequencing-and-analysis/tb-arc-project-tuberculosis-antibiotic-resistance-catalog-project (April 11 2023 2023, date last accessed).
